# Role of Multi-Detector Computed Tomography in the Diagnosis of Intestinal Obstruction

**DOI:** 10.7759/cureus.33730

**Published:** 2023-01-13

**Authors:** Shaista Afzal, Furqan Ahmad, Fizza Farooq

**Affiliations:** 1 Diagnostic Radiology, Aga Khan University Hospital, Karachi, PAK

**Keywords:** abdominal distension, specificity, sensitivity, multi-detector computed tomography, intestinal obstruction

## Abstract

Introduction: There is a need to identify patients whose small bowel obstruction (SBO) can resolve spontaneously so that unnecessary surgical interventions are avoided. This study aimed to evaluate the diagnostic accuracy of multi-detector computed tomography (MDCT) in intestinal obstruction and find out the presence, level, causes, and degree of intestinal obstruction taking intraoperative findings as gold standard.

Methodology: This cross-sectional study was conducted analyzing 147 patients that were referred from emergency with abdominal pain, abdominal distension, inability to pass flatus, and aged 18-70 years from both genders. Computed tomography (CT) examinations were done and findings like intestinal dilatation, evidence of mesenteric fat stranding, and area of transition between the dilated and collapsed loops were noted. The final report was made by the radiologist while the operative findings were reviewed from the operative notes written by operative surgeons of the same patient.

Results: In a total of 147 patients, mean age was 52.38±16.01 years. There were 76 (51.70%) males and 71 (48.30%) females. Sensitivity, specificity, positive predictive value, negative predictive value, and diagnostic accuracy of multi-detector computed tomography scan in diagnosing intestinal obstruction, taking operative findings as gold standard, were 98.39%, 65.22%, 93.85%, 88.24%, and 93.20%, respectively.

Conclusion: The multi-detector CT can be used routinely as a prime modality for detecting intestinal obstruction which will result in proper and timely management for reducing the morbidity and mortality of these particular patients.

## Introduction

Of all the patients presented in emergency department with the complaint of abdominal pain, around 15% patients have intestinal obstruction and it accounts for approximately 20% emergency surgical procedures [1-3]. Strangulation and bowel necrosis caused by small bowel obstruction is a serious condition that requires surgical intervention [4]. Intestinal obstruction resolves in about 80% of cases spontaneously with conservative management [5]. It is required to identify those patients whose small bowel obstruction (SBO) can resolve spontaneously so that unnecessary surgical interventions are avoided [6]. Around 80% cases of intestinal obstruction (IO) are found to have small bowel obstruction [7,8]. Out of total patients presented with acute abdominal pain, for hospital admissions, SBO is a significant cause of morbidity and mortality responsible for 12-16% of hospitalization in the United States [9].

The findings of this study were thought to help us in improving the diagnosis of patients coming to healthcare facilities with abdominal pain and distension who are suspected to have an intestinal obstruction. Our findings were also thought to assist us in reducing unnecessary exploratory laparotomies and unnecessary prolonged hospital stays. This study also aimed to help in timely decision and management of the patient. All these management decisions are important to reduce cost of health for patients, hospitals, and treating physicians. Our objective was to evaluate the diagnostic accuracy of multi-detector computed tomography (MDCT) in intestinal obstruction taking intraoperative findings as gold standard.

## Materials and methods

This cross-sectional study was conducted at the Department of Radiology, Aga Khan University Hospital Karachi, Pakistan, from August 2020 to February 2021. The sample size of 147 was calculated considering prevalence of intestinal obstruction of 15%, sensitivity of 94%, specificity of 90%, and margin of error of 10% for specificity and 5% for sensitivity with a confidence level of 95% [2]. Inclusion criteria were all patients that were referred to radiology department from emergency with abdominal pain, abdominal distension, and inability to pass flatus. We also included patients visiting outpatient department who had clinically presented with diagnosis of intestinal obstruction. Patients of both genders aged 18-70 years with a minimum duration of symptoms of 6 hours were enrolled. Exclusion criteria were pregnant females, abnormal kidney functioning, or patients with incomplete medical records. Approval was acquired from the ethical review committee of the study institution (reference number 2020-0669-10376). Written and informed consent was sought from all study participants.

All patients underwent CT examination. All the images were acquired on 128 slice GE Revolution Evo ES CT Scanner (Chicago, IL: GE HealthCare), after administration of intravenous contrast. All the images' acquisition was at the porto-venous phase. No oral contrast was administered. CT x-ray source parameters were as follows: x-ray tube voltage was 120.0 kV and x-ray tube current was variable and auto-selected by the CT machine according to the patient's body habitus and ranged between 200 and 350 mA. Slice thickness as 5 mm. The field of view for all the studies was from the xiphisternum to the pubic symphysis. CT feature for intestinal obstruction was small bowel diameter of 3 cm, large bowel diameter was 6 cm, and focal point of intestinal narrowing with distal intestinal luminal collapse. Signs of ischemia/necrosis like pneumatosis, portal venous gas and bowel hypoenhancement, and pneumoperitoneum were also evaluated initially but not considered in the final evaluation as these imaging findings only present when the intestinal obstruction is prolonged and making diagnosis of the intestinal obstruction become delayed leading to the compromised blood supply and perfusion to the intestinal loops leading to the ischemic changes. As these findings were not identified in all the enrolled cases and in our study, most cases presented from the emergency department or were present in the consultation clinic fulfilling inclusion/exclusion criteria. None of the patients enrolled in the study had prolonged neglected symptoms or presented from the ward setting. Only hazy/streaky mesentery was considered in the final evaluation as it was a frequent sign in cases of intestinal obstruction. The CT findings that were included in the study were intestinal dilatation, evidence of mesenteric fat stranding, and an area of transition between the dilated and collapsed loops. Final report was made by radiologist with minimum five of years post training experience. The operative findings were reviewed by the operative notes written by operative surgeons of the same patient. Operative surgeons had minimum of five years of experience. Intraoperative findings included dilated bowel loops, bowel necrosis, and intraperitoneal free fluid.

Abdominal pain was defined according to the following activities of daily living criteria and patients with moderate-to-severe type of pain were included: 0 as no pain; 1-3 as mild pain (nagging, annoying, interfering little with activities of daily living {ADLs}); 4-6 as moderate pain (interferes significantly with ADLs); 7-10 as severe pain (disabling; unable to perform ADLs). Decompressed colon was labeled when no gas and fecal matter density within the large bowel and bowel walls were opposing each other. Gasless abdomen was termed when no gas density was seen within the small bowel lumen. String-of-beads sign was labeled as small pockets of gas within a lumen of fluid-filled small bowel. Transition point was named as sudden change in calibre of the bowel lumen from dilated (>6 cm luminal diameter) to collapsed bowel lumen [10]. Abdominal distension was labeled as interval increase in abdominal diameter, measured by measuring tape at the same level of abdomen, due to any abdominal pathology. Hazy/streaky mesentery was labeled as slightly increased density and nodular appearance of the mesentery just adjacent to the inflamed bowel. Bowel necrosis was when bowel infarction or gangrenous bowel representing an irreversible injury to the intestine, secondary to compromised blood supply. The following computed tomographic criteria were used to describe intestinal obstruction: (1) major intestinal obstruction - (A) small bowel dilated to 3 cm or greater and colon not dilated (<6 cm) and (B) transition point from dilated to non-dilated small bowel; (2) minor intestinal obstruction - (A) air-fluid levels, (B) colon decompressed, (C) gasless abdomen: gas within the small bowel is a function of vomiting, and (D) string-of-beads sign as shown by small pockets of gas within a fluid-filled small bowel [11].

Diagnosis was confirmed by peroperative findings of intestinal obstruction. Diagnosis of point of obstruction and/or intestinal obstruction as per MDCT scan findings guided surgical decisions. Furthermore, clinical assessment and preferences of the consultant surgeon on duty helped in deciding whether to go for the surgery or not. Intraoperative findings included dilated bowel loops which may be due to the adhesion formations or strictures. Intraoperatively, bowel necrosis may also be noted which may be secondary to the prolonged bowel obstruction which leads to comprise in blood supply and hence bowel necrosis.

The criteria for positive and negative diagnoses are discussed as follows: true positive (TP) - presence of computed tomographic evidence of intestinal obstruction, confirmed by peroperative laparotomy findings. False positive (FP) - computed tomographic findings suggestive of intestinal obstruction, however, no peroperative evidence of intestinal obstruction. True negative (TN) - patient was admitted with suspicion of small bowel obstruction but there is no evidence of obstruction on computed tomographic, and on exploratory laparotomy other cause is identified for the presenting symptoms. False negative (FN) - patient was admitted with suspicion of intestinal obstruction but no evidence of obstruction on computed tomography, however, patient was operated on to rule out suspicion and intestinal obstruction is present.

For data analysis, SPSS version 26.00 (Armonk, NY: IBM Corp.) was used. Categorical data were represented as frequency and percentages while mean and standard deviation were calculated for continuous data. Diagnostic accuracy and sensitivity, specificity, positive predictive value (PPV), and negative predictive value (NPV) of MDCT were calculated. Effect modifiers were controlled with stratification of study variables applying chi-square test while p<0.05 was taken as significant.

## Results

**Figure 1:**
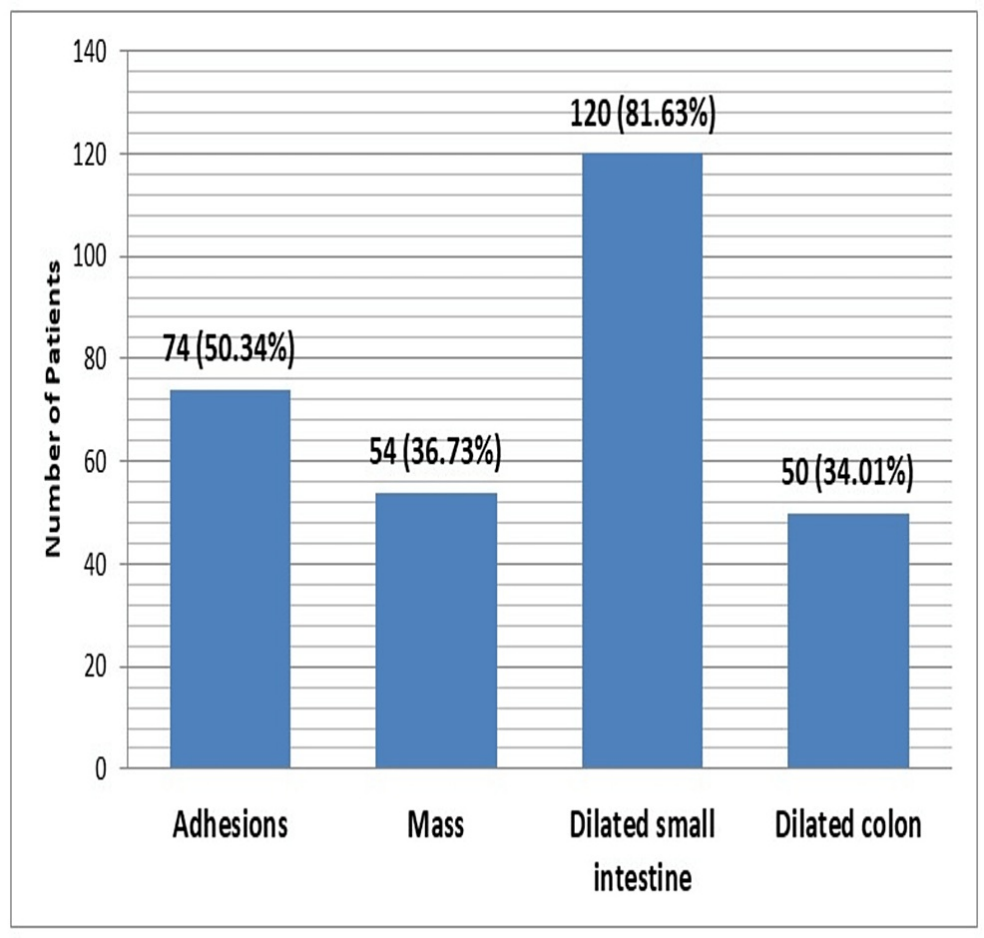
Frequency distribution of preoperative findings (n=147).

**Figure 2:**
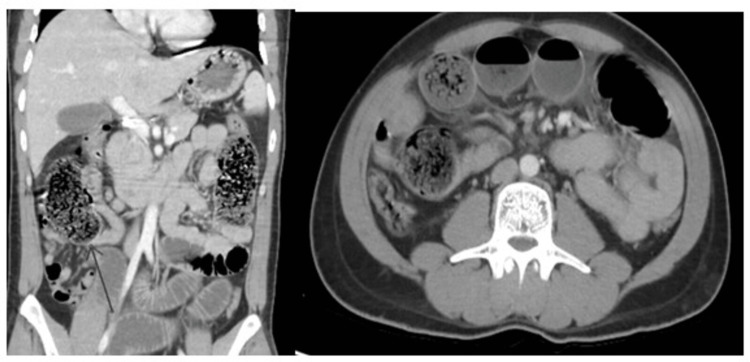
Abrupt cut of small bowel at distal ileum with dilated fecal loaded as well as fluid-filled small bowel loops proximally and collapsed terminal ileum and large bowel distally. Peroperative findings and histopathology confirm benign stricture.

**Figure 3:**
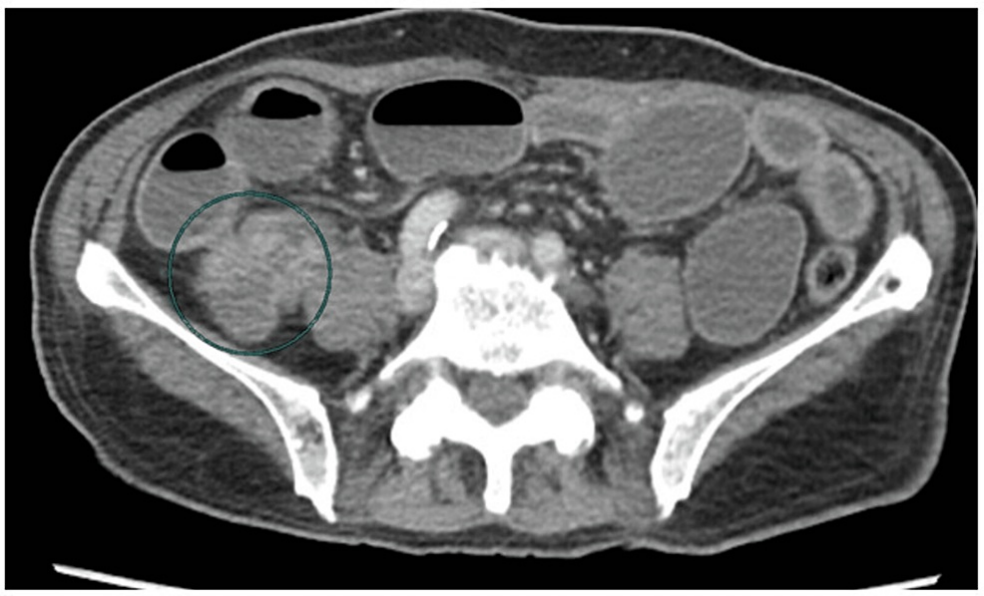
Diffusely dilated ileal loops with thickened and deformed terminal ileum and cecum CT diagnosis was likely ileocecal tuberculosis and postoperative histopathological finding was non-specific inflammation.

In a total of 147 patients, age range was 18-70 years with mean age of 52.38±16.01 years. Majority (95 {64.63%}) of the patients were between 46 and 70 years of age, while 52 (35.37%) patients had ages between 18 and 45 years. There were 76 (51.70%) males and 71 (48.30%) females with male to female ratio of 1.2:1. Mean duration of disease was 12.86±2.89 days. The mean BMI was 22.49±3.57 kg/m^2^ while 105 (71.43%) patients had BMI below 25 kg/m^2^ and remaining 42 (28.57%) had BMI ≥25 kg/m^2^. There were 143 (97.27%) patients who presented with abdominal pain while abdominal distension was observed in 122 (82.99%) patients. Frequency distributions of CT findings showed that small bowel dilated ≥3 cm, colon decompressed, hazy/streaky mesentery look, colon not dilated <6 cm, and visualization of mass were observed among 125 (85.03%), 90 (61.22%), 82 (55.78%), 90 (61.22%), and 50 (34.01%) patients, respectively. Figure 1 is showing frequency distribution of pre-operative findings. Figures 2, 3 are showing some radiological findings.

The MDCT scan supported the diagnosis of intestinal obstruction in 130 (88.44%) patients and no intestinal obstruction in 17 (11.56%) patients. Operative findings confirmed IO in 124 (84.35%) patients and no IO in 23 (15.65%) patients. In MDCT-positive patients, 122 (TP) patients had IO, and eight (FP) had no IO on peroperative findings. Among 17 patients, MDCT negative patients, two (FN) had IO on operative findings whereas 15 (TN) had no IO on operative findings (p<0.0001) (Table 1). Overall sensitivity, specificity, PPV, NPV, and diagnostic accuracy of MDCT scan in diagnosing IO, taking operative findings as gold standard, were 98.39%, 65.22%, 93.85%, 88.24%, and 93.20%, respectively. Stratification of diagnostic accuracy with respect to age groups, gender, disease duration, and BMI are shown in Tables 2-5.

**Table 1:** Diagnostic accuracy of MDCT scan in diagnosing intestinal obstruction, taking operative findings as gold standard (n=147). MDCT: multi-detector computed tomography

Variables	Positive result on operative findings	Negative result on operative findings	p-Value
Positive result on MDCT	122 (98.39%)	08 (34.78)	<0.0001
Negative result on MDCT	2 (1.61%)	15 (65.22%)	

**Table 2:** Stratification of age with respect to MDCT findings and peroperative findings (n=147). MDCT: multi-detector computed tomography; TP: true positive; FP: false positive; FN: false negative; TN: true negative; PPV: positive predictive value; NPV: negative predictive value

Variables	Positive result on operative findings	Negative result on operative findings	p-Value	Positive result on operative findings	Negative result on operative findings	p-Value
	Age between 18 and 45 years			Age between 46 and 70 years		
Positive result on MDCT	44 (95.65%)	03 (50.0%)	0.0003	78 (100%)	5 (41.67%)	<0.0001
Negative result on MDCT	02 (4.35%)	03 (50.0%)		-	12 (58.33%)	
Sensitivity	95.65%			100.0%		
Specificity	50.0%			70.59%		
PPV	93.62			93.98%		
NPV	60.0%			100%		
Diagnostic accuracy	90.38%			94.74%		

**Table 3:** Stratification of gender, with respect to MDCT findings and peroperative findings (n=147). MDCT: multi-detector computed tomography; TP: true positive; FP: false positive; FN: false negative; TN: true negative; PPV: positive predictive value; NPV: negative predictive value

Variables	Positive result on operative findings	Negative result on operative findings	p-Value	Positive result on operative findings	Negative result on operative findings	p-Value
	Male			Female		
Positive result on MDCT	64 (99.01%)	5 (45.45%)	<0.0001	58 (98.30)	3 (33.33%)	<0.0001
Negative result on MDCT	1 (0.99%)	6 (54.55%)		1 (1.70%)	9 (66.67%)	
Sensitivity	98.46%			98.31%		
Specificity	54.55%			75.0%		
PPV	92.75%			95.08%		
NPV	85.71%			90.0%		
Diagnostic accuracy	92.11%			94.37%		

**Table 4:** Stratification of duration of disease with respect to MDCT findings and peroperative findings (n=147). MDCT: multi-detector computed tomography; TP: true positive; FP: false positive; FN: false negative; TN: true negative; PPV: positive predictive value; NPV: negative predictive value

Variables	Positive result on operative findings	Negative result on operative findings	p-Value	Positive result on operative findings	Negative result on operative findings	p-Value
	Duration of disease ≤15 days			Duration of disease >15 days		
Positive result on MDCT	117 (100%)	2 (50.0%)	<0.0001	5 (71.42%)	6 (31.58%)	0.0681
Negative result on MDCT	-	2 (50.0%)		2 (25.57%)	13 (68.42%)	
Sensitivity	100.0%			71.43%		
Specificity	50.0%			68.42%		
PPV	98.32%			45.45%		
NPV	100.0%			86.67%		
Diagnostic accuracy	98.35%			69.23%		

**Table 5:** Stratification of BMI with respect to MDCT findings and peroperative findings (n=147). MDCT: multi-detector computed tomography; TP: true positive; FP: false positive; FN: false negative; TN: true negative; PPV: positive predictive value; NPV: negative predictive value

Variables	Positive result on operative findings	Negative result on operative findings	p-Value	Positive result on operative findings	Negative result on operative findings	p-Value
	BMI <25 kg/m^2^			BMI ≥25 kg/m^2^		
Positive result on MDCT	87 (98.86%)	6 (35.29%)	0.0001	35 (97.22%)	2 (33.3%)	0.0001
Negative result on MDCT	1 (1.14%)	11 (64.70%)		1 (2.78%)	4 (66.67%)	
Sensitivity	98.36%			97.22%		
Specificity	64.71%			66.67%		
PPV	93.55%			94.59%		
NPV	91.67%			80.0%		
Diagnostic accuracy	93.33%			92.86%		

## Discussion

We noted sensitivity, specificity, positive predictive value, negative predictive value, and diagnostic accuracy of MDCT scan in diagnosing intestinal obstruction, taking operative findings as gold standard, were 98.39%, 65.22%, 93.85%, 88.24%, and 93.20%, respectively. Several studies showed significance of CT in diagnosing, i.e., site, level, and the cause of intestinal obstruction with sensitivity of 94-100% and accuracy of 90-95% [12-15]. A study showed CT scan has greater efficacy in detecting small bowel obstruction with sensitivity as high as 93% and specificity of 100% with accuracy of around 94%, in diagnosing small bowel obstruction [16]. A regional study conducted in neighboring country showed sensitivity and specificity of CT scan are superior to conventional radiographs and other conventional radiographic studies, in determining bowel obstruction, along with the site and cause of obstruction [17]. But it was demonstrated that MDCT has high sensitivity for high-grade obstruction but low sensitivity for low-grade obstruction. Use of MDCT can assist in correct diagnosis of intestinal obstruction as has been shown previously [16]. Some other case series studies support our findings that MDCT has high diagnostic accuracy for IO diagnosis [18-20]. Idris et al. found MDCT to successfully detect 90% cases of SBO but 10% cases of transition were missed by MDCT [20].

A CT scan can represent a significant undertaking in a developing country like Pakistan where most healthcare facilities are resource constricted. Initially, for many decades, conventional radiological methods ranging from conventional radiography to barium studies were used for acute abdominal pain and to rule out intestinal obstruction, for which previous studies showed that conventional radiography has sensitivity of 69% and specificity of 57% [21]. Some researchers have revealed MDCT to be valuable in diagnosing site, level, and the cause of intestinal obstruction showing its sensitivity ranging between 94% and 100% while the overall accuracy ranged between 90% and 95% [22]. There are some suggestions that CT scan has more significant role in deciding the cause and/or severity of obstruction instead of diagnosing it.

One of the major aims of prompt diagnosis of intestinal obstruction is to avoid complications like ischemia and bowel necrosis [23]. Usually, the diagnosis of bowel obstruction is made through history, clinical examination, and radiological findings but plain radiographs are known to have low sensitivity, specificity and accuracy stated in the literature as 69%, 57%, and 46-80% respectively [17,24]. As per the findings of this study, MDCT has high sensitivity and specificity in diagnosing small bowel obstruction. Furthermore, the MDCT not only identifies the site of obstruction, but it can also assist in finding out the cause of obstruction as has been found by other researchers as well [25].

Diagnostic laparotomy is considered a common practice in our part of the world for suspected intestinal obstruction while through this study, we wanted to highlight the significance of MDCT in helping towards the right diagnosis among children coming to us with abdominal pain, abdominal distension, and inability to pass flatus [26]. As not much local data exists regarding the role of MDCT in the diagnosis of intestinal obstruction, the present study seems to be a worthy addition to whatever local data exists in this regard.

Limitations of this study

Being a single-center study noting only immediate outcomes were the limitations of this study. The sample size of the current research was relatively small.

## Conclusions

The MDCT scan was a highly sensitive and accurate non-invasive modality in detecting intestinal obstruction and had not only dramatically improved our ability of accurate diagnosis of intestinal obstruction but also improved patient care through timely and proper treatment. The MDCT can be used routinely as a prime modality for detecting intestinal obstruction which will result in proper and timely management for reducing the morbidity and mortality of these particular patients.
